# THE ROLE OF IMMUNOINFLAMMATORY MARKERS IN THE PROGNOSIS AND RESECTABILITY OF PANCREATIC ADENOCARCINOMA

**DOI:** 10.1590/0102-672020180001e1366

**Published:** 2018-07-02

**Authors:** Tatiana Falcão EYFF, Henrique Rasia BOSI, Mariana Sandrin TONI, Mariana Blanck ZILIO, Carlos Otavio CORSO, Vivian Pierri BERSCH, Alessandro Bersch OSVALDT

**Affiliations:** 1 Programa de Pós-graduação em Ciências Cirúrgicas, Faculdade de Medicina, Universidade Federal do Rio Grande do Sul; 2 Serviço de Cirurgia do Aparelho Digestivo, Hospital de Clínicas de Porto Alegre); 3 Grupo do Pâncreas, Hospital Moinhos de Vento, Porto Alegre, RS, Brazil.

**Keywords:** Carcinoma, pancreatic ductal, Prognosis, Inflammation mediators., Carcinoma ductal pancreático, Prognóstico, Mediadores da inflamação

## Abstract

***Background*:**

Pancreatic adenocarcinoma has a high mortality rate. A prognostic tool is essential for a better risk stratification. The neutrophil/lymphocyte ratio and adaptations and the platelet/lymphocyte ratio seem promising for this purpose.

***Aim*:**

Evaluate the prognostic value of neutrophil/lymphocyte ratio, derived neutrophil/lymphocyte ratio and platelet/lymphocyte ratio, analyze the ideal cutoff values and investigate their utility in predicting resectability.

***Methods*:**

Data were collected of patients with pancreatic adenocarcinoma in Hospital de Clínicas de Porto Alegre between 2003 and 2013. The studied ratios were determined by blood count collected at hospital admission and after two cycles of palliative chemotherapy.

***Results*:**

Basal neutrophil/lymphocyte ratio, derived neutrophil/lymphocyte ratio and platelet/lymphocyte ratio did not have prognostic impact in survival (p=0.394, p=0.152, p=0.177 respectively). In subgroup analysis of patients submitted to palliative chemotherapy, neutrophil/lymphocyte ratio, derived neutrophil/lymphocyte ratio and platelet/lymphocyte ratio determined after two cycles of chemotherapy were prognostic for overall survival (p=0.003, p=0.009, p=0.001 respectively). The ideal cutoff values found were 4,11 for neutrophil/lymphocyte ratio (sensitivity 83%, specificity 75%), 2,8 for derived neutrophil/lymphocyte ratio (sensitivity 87%, specificity 62,5%) and 362 for platelet/lymphocyte ratio (sensitivity 91%, specificity 62,5%), Neutrophil/lymphocyte ratio, derived neutrophil/lymphocyte ratio and platelet/lymphocyte ratio were not able to predict resectability (p=0.88; p=0.99; p=0.64 respectively).

***Conclusions*:**

Neutrophil/lymphocyte ratio, derived neutrophil/lymphocyte ratio and platelet/lymphocyte ratio are useful as prognostic markers of overall survival in patients with pancreatic adenocarcinoma submitted to palliative chemotherapy. Its use as resectability predictor could not be demonstrated.

## RESUMO

***Racional*:**

O adenocarcinoma pancreático apresenta alta taxa de mortalidade. Uma ferramenta que possa predizer adequadamente o seu prognóstico é fundamental para melhor estratificação de risco. A razão neutrófilos/linfócitos e suas adaptações e a razão plaquetas/linfócitos tem se mostrado promissores para este fim.

***Objetivo*:**

Avaliar o valor prognóstico das razões neutrófilos/linfócitos, neutrófilos/linfócitos derivada e plaquetas/linfócitos, analisar os pontos de corte mais adequados e investigar sua utilidade como fator preditivo de ressecabilidade.

***Métodos*:**

Foram coletados dados de pacientes com adenocarcinoma pancreático atendidos no Hospital de Clínicas de Porto Alegre entre 2003 e 2013. As razões estudadas foram determinadas com base nos hemogramas coletados na internação e após dois ciclos de quimioterapia paliativa.

***Resultados*:**

As razões neutrófilos/linfócitos basal, neutrófilos/linfócitos derivada basal e plaquetas/linfócitos basal não tiveram impacto prognóstico na sobrevida (p=0,394, p=0,152, p=0,177 respectivamente). No subgrupo submetido a quimioterapia paliativa, as razões neutrófilos/linfócitos, neutrófilos/linfócitos derivada e plaquetas/linfócitos após dois ciclos de tratamento mostraram-se fatores prognósticos para sobrevida global (p=0,003, p=0,009 e p=0,001 respectivamente). Os pontos de corte encontrados foram 4,11 para neutrófilos/linfócitos (sensibilidade 83% e especificidade 75%), 362 para plaquetas/linfócitos (sensibilidade 91% e especificidade 62,5%) e 2,8 para neutrófilos/linfócitos derivada (sensibilidade 87% e especificidade 62,5%). As razões neutrófilos/linfócitos, neutrófilos/linfócitos derivada e plaquetas/linfócitos não se mostraram estatisticamente significativas como preditores para ressecabilidade (p=0,88; p=0,99 e p=0,64 respectivamente).

***Conclusões*:**

As razões neutrófilos/linfócitos, neutrófilos/linfócitos derivada e plaquetas/linfócitos são úteis como marcadores prognósticos de sobrevida global em pacientes com adenocarcinoma pancreático submetidos à quimioterapia paliativa. Seu uso como preditor de ressecabilidade não foi demonstrado.

## INTRODUCTION

Pancreatic adenocarcinoma (PAC) accounts for more than 90% of pancreatic neoplasias² and is one of the most lethal tumors¹. In Brazil PAC accounts for about 2% of all cancers diagnosed and 4% of all cancer deaths^16^. This disease is associated with an extremely poor prognosis, reflected by a 5-year survival of less than 5% when all stages are combined^9^. 

Surgical resection is the only form of curative treatment^18^, but at the moment they are diagnosed, only 20% of PAC patients are eligible for a resection^22^. Diagnosis at a late stage is one of the main reasons for the low treatment success rates^15^. However, even after curative surgical resection, many patients will present relapse and the 5-year survival of patients with complete resection is only about 25%^13^. Unfortunately, although precursor lesions that may progress into an invasive adenocarcinoma have already been determined^2^ ^,^ ^3^ ^,^ ^15^ ^,^ ^22^ ^,^ ^26^, the existing techniques for identifying them in the general population are not feasible due to their high costs, low incidence of disease and the difficult location of the organ (retroperitoneal)². Thus, at the time of diagnosis, most patients will have palliative chemotherapy or clinical support as their only treatment option^21^.

It is important to develop a prognostic tool that can predict the survival of patients and allow the tailoring of therapies for patients who would have a worse outcome^18^. The identification of new prognostic factors allows a better risk stratification for adjuvant treatments after surgical resection or more aggressive treatments in patients with metastatic disease^13^.

In recent years, evidence has emerged showing that the systemic inflammatory response could play an important role in the development and progression of various types of cancer^12^, being closely linked to the prognosis^13^. 

The neutrophil-to-lymphocyte ratio (NLR) is attracting increasing attention^14^. The association between elevated NLR and a poor prognosis after resection or chemotherapy in a variety of cancers has been demonstrated. In PAC, several studies have also shown this association^29^. Most of them suggest that NLR represents a factor of poor prognosis when above 5^14^ ^,^ ^18^ ^,^ ^21^ ^,^ ^29^, but other cutoff values have already been shown to be effective^5^ ^,^ ^10^ ^,^ ^11^ ^,^ ^20^ ^,^ ^30^.

Adaptations and derivations of NLR have been developed aiming to increase its use or improve its prognostic accuracy. One example is the derived neutrophil-to-lymphocyte ratio (dNLR=neutrophil count/[total leukocytes - neutrophils]), published by Proctor et al in 2012^17^. This study showed the use of dNLR as a prognostic factor in 700 patients with tumors of the liver, pancreas and bile ducts, using a cutoff point of 2.5. In 2013, Szkandera et al conducted a study for the external validation of dNLR as a prognostic factor in patients with pancreatic cancer, showing that this is an independent factor for specific survival when above 2.3 (HR 1.24, CI 95%=1.01-1.51, p=0.041)^24^. Another study has also developed a prognostic score based on the NLR in the blood collected within one week before the start of chemotherapy and the difference in NLR after one cycle of chemotherapy. A worse prognosis was demonstrated the higher the basal NLR was and the lower the decrease in this rate after one cycle of chemotherapy^6^.

The platelet-to-lymphocyte ratio (PLR) is another index based on inflammatory parameters. Its prognostic value has already been studied in tumors of different types with controversial results. In 2014, a meta-analysis was published that concluded that PLR can act as a prognostic factor in several types of cancer, using a cutoff point that varied from 150 to 300^28^. Its performance has already been evaluated specifically in patients with advanced PAC, being useful as a prognostic factor using a cutoff point of 200 (PLR<200: overall survival 9.1 months vs. 4 months if PLR>200, p=0.007)^14^.

The objective of the present study was to evaluate the prognostic value of the NLR, dNLR and PLR determined by blood count collected at admission and after palliative chemotherapy in a population of patients diagnosed with pancreatic adenocarcinoma, also analyzing the appropriate cutoff value for each parameter. In addition, was the intend to investigate whether these ratios may have some value as a predictive factor for resectability in pancreatic adenocarcinoma.

## METHODS

After approval of the study by the Research Ethics Committee (GPPG 14028-3), patients with pancreatic adenocarcinoma that had been treated at Hospital de Clínicas de Porto Alegre (HCPA) with a diagnosis between January 2003 and December 2013 were selected. Data were collected retrospectively through the electronic medical record system. The following terms were used through the International Classification of Diseases (ICD) to search for patients in the electronic medical record: ICD C25.0 (malignant neoplasm of head of pancreas), ICD C25.1 (malignant neoplasm of body of pancreas), ICD C25.2 (malignant neoplasm of tail of pancreas), ICD C25.3 (malignant neoplasm of pancreatic duct), ICD C25.7 (malignant neoplasm of other parts of pancreas), ICD C25.8 (malignant neoplasm of overlapping sites of pancreas) and ICD C25.9 (malignant neoplasm of pancreas, unspecified). Patients who did not have the diagnosis confirmed by histopathological examination were excluded.

All researchers involved in the study and data collection signed a Term of Commitment for Data Use, ensuring the privacy and anonymity of patients. Demographic data, information on symptoms, imaging tests, laboratory tests, treatments performed and dates of death were collected. Were registered the data from the blood counts collected at hospital admission, before surgery or the start of chemotherapy treatment and after two cycles of palliative chemotherapy. All cases were reclassified according to the 2010 American Joint Committee on Cancer (AJCC) staging. For patients who did not have a death registry in the HCPA system, the data were referred to the Núcleo de Informações em Saúde (Health Information Center) of the state’s Health Department for the confirmation of the dates of the deaths. For patients whose death dates were not found in any of these systems, the date of the last service recorded at the HCPA was used.

### Statistical analysis

The database was created and analyzed in the version 20.0 of the statistical program IBM SPSS. The quantitative variables were described as mean and standard deviation when of normal distribution or as median, minimum and maximum. Categorical variables were presented as absolute or relative frequencies. The level of significance was set at 5% (p<0.05). The Kaplan-Meier method was used for the survival analysis and the differences were compared through Log-Rank. The Cox regression was used to evaluate prognostic factors. The analysis of cutoff points was performed using a ROC curve. The resectability evaluation was performed through Poisson regression with robust variances. The neutrophil-to-lymphocyte ratio (NLR) was determined by the neutrophil count divided by the lymphocyte count of the same blood count, the platelet-to-lymphocyte ratio (PLR) was calculated by the platelet count divided by the lymphocyte count and the derived neutrophil-to-lymphocyte ratio (dNLR) was defined by the following equation: neutrophils / (total leukocytes - neutrophils). The difference in post-palliation NLR (Dif NLR) was determined by the calculation of the NLR after palliation - pre-treatment NLR. The post-palliation score groups were defined as the sum of the following scores: basal NLR (score 0, NLR <2.5, score 1, NLR 2.5-4.4, score 2, NLR> 4.4) and Dif NLR (score 0: DifNLR <0; score 1: DifNLR>0). Overall survival was calculated as the time elapsed from the date of diagnostic confirmation by histopathological examination to the date of death for any cause (n=119) or censored as the date of the last evaluation (n=16).

## RESULTS

One hundred and thirty-five patients diagnosed with pancreatic adenocarcinoma confirmed by histopathological examination were selected. Among these, 70 were women (51.9%) and 65 were men (48.1%). The mean age at diagnosis was 64.11+11.41 years. History of smoking was present in 43.7% of the patients, alcoholism in 15.6% and diabetes mellitus in 26.7%. Among the symptoms reported, the most common was weight loss (75.6%), followed by abdominal pain (66.7%), jaundice (58.5%), coluria (50.4), and acholia (41.5%). Other symptoms reported were anorexia (39.3%), nausea (29.6%), vomiting (23.7%), feeling full (11.1%), pruritus (25.9%), tiredness (17.8%), weakness (16.3%) and dyspnea (3.7%). Cholangitis occurred in 6.7% of patients and acute pancreatitis in 5.9%. The mean weight loss at the time of diagnosis was 9.2+7.8 kg, the mean BMI at diagnosis was 24+4.5 kg/m².

In most patients (79%) the tumor was located in the head or uncinate process of the pancreas. Based on the imaging tests available in the electronic medical record (n=109), the patients were reclassified by AJCC 2010 in stage IA (12.6%), IB (8.1%), IIA (14.8%), IIB (12.6%), III (6.7%) and IV (25.9%). Regarding the treatments received, 84 patients were operated, of which 48 (57.1%) had resection with curative intent and 36 (42.9%) underwent palliative or merely diagnostic surgery. Adjuvant chemotherapy was started at HCPA for 19 patients (39.6% of patients undergoing curative resection), while 49 patients received palliative chemotherapy (22 considered primarily unresectable, 21 submitted to palliative/diagnostic surgery, and six patients who underwent curative resection with recurrence).

Data for calculating the neutrophil-to-lymphocyte and derived neutrophil-to-lymphocyte ratios at the time of admission (basal NLR and basal dNLR) were available for 126 patients, while the basal platelet-to-lymphocyte ratio calculation was possible for 110 patients. Among the patients who had undergone palliative chemotherapy, 31 had blood counts after two cycles of chemotherapy. The median basal NLR was 3.04 (0.8-23), the median basal dNLR was 5.09 (1.18-30.15) and the median basal PLR was 161.77 (44.6-662.8). After two cycles of treatment with palliative chemotherapy the median NLR was 3.1 (0.38-15.92), the median dNLR was 2.04 (0.29-7.34) and the median PLR was 225.38 (70.25-1622.8). There was a decrease in NLR after palliative chemotherapy in 10 of the patients submitted to treatment (32.3%). The demographic, clinical and laboratory characteristics, as well as the treatments performed can be seen in Table 1.

**TABLE 1:** Patients characteristics

Parameter		Value
Clinical and demographic characteristics )	n	%
Age (years)a		64.1+11.4
Gender		
Female	70	51.9%
Male	65	48.1%
Smoking	59	43.7%
Alcoholism	21	15.6%
Diabetes	36	26.7%
ECOG status (n=82)		
0	8	9.8%
1	43	52.4%
2	16	19.5%
3	12	14.6%
4	3	3.7%
Symptoms (n=135)	n	%
Weight loss	102	75.6%
Lost weight (kg) - (n=78)a		9.2+7.9
BMI (kg/m²) - (n=83)a		24.1+4.5
Abdominal pain	90	66.7%
Jaundice	79	58.5%
Colúria	68	50.4%
Acholia	56	41.5%
Pruritus	35	25.9%
Nausea	40	29.6%
Vômiting	32	23.7%
Feeling full	15	11.1%
Anorexia	53	39.3%
Tiredness	24	17.8%
Weakness	22	16.3%
Dyspnea	5	3.7%
Cholangitis	9	6.7%
Pancreatitis	8	5.9%
Tomographic characteristics	n	%
Tumor size (cm) - (n=78)a		3.99+2.01
Location (n=98)		
Head/uncinate process	79	80.6%
Body/tail	19	19.4%
Vascular invasion (n=105)		
Celíac axis	7	6.7%
Superior mesenteric artery	7	6.7%
Hepatic artery	2	1.9%
Superior mesenteric vein	8	7.6%
Lymph node involvment (n=106)	47	44.3%
Metastasis (n=110)	33	30%
Staging (n=109)		
IA	17	15.6%
IB	11	10.1%
IIA	20	18.3%
IIB	17	15.6%
III	9	8.3%
IV	35	32.1%
Laboratory tests - basal		Mediana (mín-máx)
Hemoglobin (g/dl) - (n=127)a		12.2+1.9
Total leukocytes (x 10³/µl) - (n=126)		7.81 (2.09-34.26)
Lymphocytes (x 10³/µl) - (n=126)		1.52 (0.54-4.32)
Neutrophils (x 10³/µl) - (n=126)		5.09 (1.18-30.15)
Platelets (x 10³/µl) - (n=126)		247 (82-613)
Total bilirubin (mg/dl) - (n=122)		9.6 (0.3-57.7)
Albumin (g/dL) - (n=100)		3.8 (1-5)
Ca 19.9 (U/ml) - (n=43)		354.9 (0.6-12530)
NLR (n=126)		3.05 (0.8-23.02)
dNLR (n=126)		5.09 (1.18-30.15)
PLR (n=110)		161.77 (44.61-662.82)
Treatment	n	%
Surgery (n=84)		
Curative resection	48	57.1%
Palliative/diagnostic	36	42.9%
Chemotherapy (n=65)		
Adjuvante	19	29.23%
Paliativa	49	75.38%
Laboratory tests - post-palliation		Mediana (min-max)
Total leukocytes (x 10³/µl) - (n=32)		7.88 (2.85-15.71)
Neutrophils (x 10³/µl) - (n=32)		5.17 (1.02-11.78)
Lymphocytes (x 10³/µl) - (n=31)		1.6 (0.57-3.28)
Platelets (x 10³/µl) - (n=32)		337.5 (117-1313)
NLR (n=31)		3.17 (0.38-15.92)
dNLR (n=31)		2.05 (0.29-7.34)
PLR (n=31)		225.39 (70.25-1622.81)
Dif NLR (n=31). n %		
< 0	10	32.3%
>0	21	67.7%
Post-palliation score group (n=31). n%		
0	3	9.7%
1	16	51.6%
2	9	29%
3	3	9.7%

aDatas presented as mean +/- standard deviation; ECOG=Eastern Cooperative Oncology Group; BMI=body mass index; Ca 19.9=carbohydrate antigen 19.9; NLR=neutrophil count/lymphocyte count; dNLR=neutrophil count/(total leukocytes - neutrophil count); PLR=platelet count/lymphocyte count; DifNLR=NLR after palliation - NLR pre-treatment

### Survival analysis

Median overall survival was five months, ranging from 0-121 months in patients who received treatment with curative intention and 0-45 months in those who received palliative or supportive care.

**FIGURE 1:**
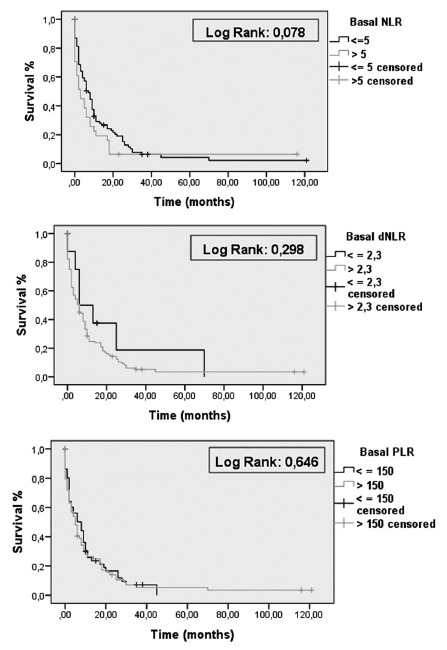
Survival analysis through Kaplan-Meier considering NLR, dNLR e PLR values at hospital admission

When analyzing the data using the Cox regression, considering the whole population included in the study, basal NLR, basal dNLR and basal PLR showed no evidence of a prognostic impact on survival (p=0.394, p=0.152, p=0.177, respectively). Similar results can be seen by using the Kaplan-Meier curves analysis, which shows no difference in survival between the different groups of NLR (<5, >5), dNLR (<2.3, >2.3) and PLR (<150, >150), using cutoff points already described in the literature (Figure 1).

When was evaluated the group of patients undergoing palliative chemotherapy, NLR, dNLR and PLR calculated by blood counts performed after two treatment cycles have shown to be prognostic factors for overall survival in the Cox regression analysis (p=0.003, p=0.009 and p=0.001 respectively, Table 2). Other factors that were analyzed were age in years, pre-treatment staging (stages I or II vs. III or IV), gender (male vs. female), weight lost in kg, BMI, tumor size in cm, tumor location (head/uncinate process vs. body/tail), albumin levels, CA 19.9 levels, ECOG status (0 or 1 vs. 2, 3 or 4), decrease in NLR after palliation and post-palliation score (0 or 1 vs. 2 or 3).

**TABLE 2:** Cox regression

Variable	HR (IC 95%)	p
Age (years)	1 (0.96-1.04)	0.9
Staging TC		
I / II		
III / IV	2.88 (1.25-6.67)	0.01
Gender		
Female	2.39 (1.1-5.16)	0.02
Male		
Weight loss (kg)	1.01 (0.97-1.05)	0.59
BMI	1.05 (0.93-1.2)	0.4
Size TC (cm)	1.05 (0.75-1.48)	0.76
Location TC		
Head/uncinate process		
Body/tail	2.18 (0.88-5.38)	0.09
Albumin	1.75 (0.48-6.36)	0.39
CA 19.9	1 (0.99-1.002)	0.94
ECOG		
0 /1		
2 /3 / 4	4.18 (1.46-11.94)	0.00
NLR post-palliation	1.28 (1.08-1.52)	0.00
PLR post-palliation	1.004 (1.002-1.006)	0.00
dNLR post-palliation	1.57 (1.09-2.28)	0.01
DifNLR		
< 0		
>0	1.87 (0.84-4.14)	0.12
Post-palliation score		
0 / 1		
2 / 3	2.72 (1.19-6.25)	0.01

HR=hazard ratio; CI 95%=confidence interval 95%; p<0,05 considered statistically significant

**FIGURE 2:**
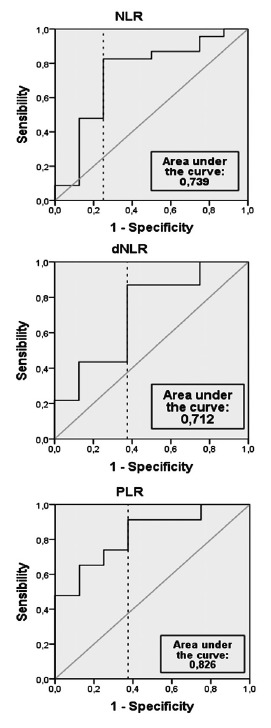
ROC Curve of 180-day survival using NLR, dNLR e PLR values after palliative treatment

To verify the most appropriate cutoff points for this population, a ROC curve with survival evaluation 180 days after diagnosis was performed. The following cutoff points were found: NLR=4.11 (sensitivity 83% and specificity 75%), PLR=362 (sensitivity 91% and specificity 62.5%) and dNLR=2.8 (sensitivity 87% and specificity 62.5%). The graphs of the curves can be seen on Figure 2.

**FIGURE 3:**
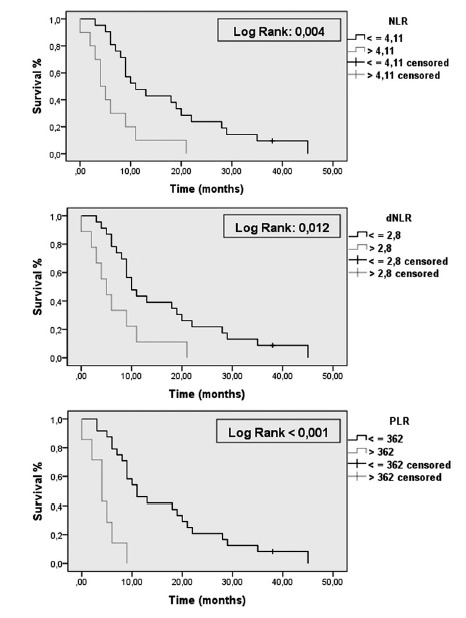
Survival analysis through Kaplan-Meier considering NLR, dNLR e PLR values after palliative treatment

Corroborating the results found, the Kaplan-Meier method was used to separate the population submitted to palliative chemotherapy in two groups according to the cutoff points found for each prognostic parameter. The result was the confirmation that there is a survival difference between the groups (NLR<4,11: overall survival 11 vs. 4 months, p=0.004, dNLR<2.8: overall survival 10 vs. 5 months, p=0.014, PLR<362: overall survival 11 vs. 4 months, p<0.001). The population survival curves can be seen on Figure 3.

### Resectability evaluation

In order to evaluate if the NLR, dNLR and PLR may have a predictive role in the resectability of pancreatic adenocarcinoma, an analysis of the patients submitted to surgery with curative intent was performed. A total of 84 patients have undergone a surgical procedure. Of these, 48 (57.1%) had the possibility of curative resection through pancreatoduodenectomy or distal pancreatectomy, while 36 (42.9%) had the intraoperative finding of the lesion to be unresectable due to being locally advanced or due to the presence of metastasis. In the Poisson regression analysis with robust variances, none of the ratios were statistically significant as a predictor for resectability (NLR, p=0.88, dNLR, p=0.99, PLR, p=0.64).

## DISCUSSION

PAC is one of the neoplasias that is most worrying due to its increasing incidence^12^ ^,^ ^22^, high morbidity and mortality^9^ , difficulty in early diagnosis^2^ ^,^ ^18^ and lack of treatment that can cause substantial improvement in long-term survival rates^2^ ^,^ ^4^ ^,^ ^27^. The identification of prognostic factors in patients with advanced disease is useful both to counsel the patient and to assist the medical team in making decisions, such as indicating a more invasive method of biliary drainage, but with a longer duration for patients with better prognosis and the opposite for those patients with estimated survival of a few weeks to a few months. In addition, there are indications that the ratios studied can be used to evaluate the response to palliative chemotherapy^10^ ^,^ ^14^, which is extremely important information in order to decide on the continuity of treatment considering its adverse reactions and time spent in hospital visits for being medicated and tested.

The present study evaluated the role of inflammatory markers, specifically the ratios NLR, dNLR and PLR, in the prognosis of patients with pancreatic adenocarcinoma. Our cohort of patients included both individuals with resectable tumors who underwent surgical treatment with curative intent, as well as patients with advanced disease undergoing palliative care and even individuals who, due to their clinical status, were only maintained on supportive care. A statistically significant difference in the overall survival of the patients undergoing palliative chemotherapy was demonstrated when using the tests performed after two cycles of chemotherapy, with the values of 4.11 for the NLR, 2.8 for dNLR and 362 for PLR being identified as the cutoff points. When all patients were analyzed together using the tests performed at the time of admission as a basis for calculation, differences in survival could not be detected. In the evaluation of the patients submitted to surgical treatment, the NLR, dNLR and PLR did not demonstrate to be useful to predict the resectability of the lesions.

A crucial factor that generated great interest for the ratios presented in this study is the ease of obtaining them through peripheral blood collection and blood count. Thus, these can become tools easily implanted, even in areas with limited resources.

The relationship between the NLR and the prognosis of malignant diseases has already been evaluated in several studies. Clark et al, in 2007, evaluated 44 patients with primarily resected pancreatic cancer and found no prognostic relevance for high NLR^7^. Another negative study including 51 primarily resected patients was reported by Sanjay et al in 2012^19^. In contrast to these studies, Wang et al found a worse prognosis in 177 pancreatic cancer patients regardless of the therapeutic strategy when there was a high NLR^25^ and Garcea et al could also prove the role of NLR as a significant prognostic parameter for disease-free survival in 74 patients with pancreatic cancer^8^. Stotz^21^, in 2013, conducted a study that aimed to validate NLR as a prognostic parameter in a large cohort of 371 patients with pancreatic cancer. High NLR was shown to be a poor prognostic factor for patients with primarily operable or metastatic pancreatic cancer^21^. In another study conducted by Xue et al in 2014^29^, NLR not only proved to be an independent factor of bad prognosis in patients with PAC undergoing chemotherapy, but also that the decrease in this value after the first cycle of chemotherapy suggested a better response to treatment. In this study, median overall survival was 12.8 months in patients who had NLR<5 and six months in those whose ratios were above 5. In addition, patients whose NLR values were >5 before the treatment and fell to <5 after the first cycle of chemotherapy had significantly longer time before treatment failure (4.3 vs. 1.4 months) and longer overall survival (9.3 vs. 2.4 months) when compared with patients whose NLR values remained >5^29^.

In 2015, Cheng et al^5^ conducted a meta-analysis evaluating elevated NLR as a poor prognostic factor for overall survival of patients with PAC. Nine articles were included and the cutoff points used to consider high NLR ranged from 2 to 5. Shorter survival was demonstrated when the NLR was above the cutoff point with a HR of 1.587 (1.411 - 1.785, p<0.01)^5^. Another meta-analysis was conducted by Yang et al^30^. The cutoff points for NLR ranged from 2.3 to 5. There was a difference in overall survival, showing a HR of 1.2 in patients submitted to surgical resection and 2.08 in patients undergoing palliative chemotherapy^30^. In our study, the ideal cutoff point was 4.1, which is similar to the values found in the previous studies.

Choi, in 2016^6^, published a retrospective study with 396 patients with metastatic PAC who received palliative chemotherapy. Blood counts within one week prior to the chemotherapy cycle and after the first cycle were evaluated. A score based on the NLR value before the treatment and whether or not that value decreased after the first cycle of chemotherapy was created. A significant difference in overall survival was observed considering the different NLR pre-treatment values (median survival NLR <2.5: 9 months, 2.5-4.4: 7.2 months,>4.4: 3.9 months) and also when evaluated considering the score (group a median survival (Score 0) 9.7 months, group b (score 1) 7.9 months, group c (score 2) 5.7 months and group d (score 3) 2.6 months)^6^. In this study, a significant difference in the overall survival was demonstrated for palliative patients when the scores were applied and groups *a* and b were compared to groups c and d, but the n of patients undergoing palliative treatment was too small for an analysis comparing each category to be performed. 

The dNLR emerged from the willingness to use databases of studies with palliative treatment. When patients enter chemotherapy studies, only the total leukocyte count and the neutrophil count are routinely recorded. In an attempt to eliminate this problem and to allow a broader use of the scores based on the inflammatory parameters in these scenarios, the derived NLR was developed. Proctor et al^17^ evaluated the prognostic role of dNLR in a large cohort of 12,118 patients with different types of cancer, including PAC, and clearly demonstrated that the dNLR has a similar effect to NLR on prognosis, showing a worse clinical outcome in patients with a high ratio and the possibility of using it to predict survival^17^. In order to validate the prognostic relevance of this marker, Szkandera et al^24^ conducted a study in 2013 investigating the prognostic value of pre-treatment dNLR in cancer-specific survival in a large cohort of PAC patients. The previously published cutoff value of 2^24^ was validated. In 2015, Suzuki et al^23^ published a study demonstrating that dNLR is also a reliable predictive marker for assessing the response to gemcitabine chemotherapy in patients with unresectable PAC. In this study, the cutoff point used was 2.5.

The PLR is probably the second most studied method based on the inflammatory parameters after the NLR. Xin Zhou et al^28^ have published a meta-analysis that assessed PLR as a prognostic factor for several types of cancer. Twenty-six studies from 10 countries that used cutoff points between 100 and 300 for PLR and tumors on several sites (breast, ovary, colorectal, esophagus, stomach, pancreas, hepatocarcinoma, pleural mesothelioma, lung, kidney) were included. The conclusion was that a high PLR predicts a worse prognosis for overall survival with a combined hazard ratio (HR) of 1.6. However, in the subgroup analysis, PLR was not prognostic for pancreatic cancer (HR=1)^28^. Martin et al^14^ evaluated PLR as a prognostic factor specifically within a population with advanced PAC. The cutoff point used for PLR was 200 and a HR=1.64 was found for high PLR with median survival of 4 vs. 9.1 months for those with PLR<200^14^. In this study, the cutoff value found through a ROC curve for PLR was 362, higher than what is usually used in the literature. 

As far as we know, this is the first study to evaluate inflammatory markers in pancreatic cancer with the Brazilian population. Another positive aspect of our study is the confirmation of the exact date of death for most patients, which makes the information more reliable. Moreover, the results were consistent even with a reduced n for the population of patients undergoing palliative chemotherapy.

Biologically, the explanation for finding a prognostic value in the ratios studied is due to an imbalance between the protective ability of the lymphocyte infiltration in the tumor bed and the effect of stimulation of proliferation caused by substances released by neutrophils and platelets. Lymphocytes play a major role in immune surveillance, which prevents tumor development^29^. Lymphopenia indicates disease severity and is linked to the immune escape of tumor cells from tumor infiltrating lymphocytes. It has been shown that higher levels of tumor infiltrating lymphocytes at the primary site is associated with a better prognosis^5^. Neutrophils, conversely, have been reported to be the primary source of circulating VEGF, which has been shown to play a crucial role in tumor angiogenesis and, therefore, has a close relationship with vascular invasion and metastasis in cancer^5^. Similar to neutrophils, platelets can release various growth factors, such as platelet-derived growth factor, platelet factor 4, TGF-beta, VEGF, and thrombospondin. These growth factors can function as potent mitogens and stimulate tumor cell proliferation and adhesion to other cells leading to tumor growth and dissemination of metastasis^29^. With this association between the presence of neutrophils and platelets and the potential for tumor dissemination, it is believed that the ratios studied may have some relation with the staging and the resectability potential of the lesions. In this study, this relationship cannot be verified. However, we believe that this possibility should be explored in future prospective studies.

## CONCLUSION

The parameters presented clearly demonstrated to predict survival differences in patients undergoing palliative chemotherapy. However, when assessing all patients together, the role of the ratios studied was not demonstrated. This may be due to the differences at the time of evaluation, with some patients already with advanced disease and others who became symptomatic near the registered care. Setting the ideal time to use these parameters for prognostic evaluation remains a challenge
